# Host sharing by the honey bee parasites *Lotmaria passim* and *Nosema ceranae*

**DOI:** 10.1002/ece3.2796

**Published:** 2017-02-15

**Authors:** Manuel Tritschler, Gina Retschnig, Orlando Yañez, Geoffrey R. Williams, Peter Neumann

**Affiliations:** ^1^ Institute of Bee HealthVetsuisse FacultyUniversity of BernBernSwitzerland; ^2^ AgroscopeSwiss Bee Research CentreBernSwitzerland; ^3^ Department of Entomology and Plant PathologyAuburn UniversityAuburnALUSA

**Keywords:** competition, interaction, *Lotmaria passim*, *Nosema ceranae*

## Abstract

The trypanosome *Lotmaria passim* and the microsporidian *Nosema ceranae* are common parasites of the honey bee, *Apis mellifera*, intestine, but the nature of interactions between them is unknown. Here, we took advantage of naturally occurring infections and quantified infection loads of individual workers (*N* = 408) originating from three apiaries (four colonies per apiary) using PCR to test for interactions between these two parasites. For that purpose, we measured the frequency of single and double infections, estimated the parasite loads of single and double infections, and determined the type of correlation between both parasites in double infections. If interactions between both parasites are strong and antagonistic, single infections should be more frequent than double infections, double infections will have lower parasite loads than single infections, and double infections will present a negative correlation. Overall, a total of 88 workers were infected with *N. ceranae*, 53 with *L. passim*, and eight with both parasites. Although both parasites were found in all three apiaries, there were significant differences among apiaries in the proportions of infected bees. The data show no significant differences between the expected and observed frequencies of single‐ and double‐infected bees. While the infection loads of individual bees were significantly higher for *L. passim* compared to *N. ceranae*, there were no significant differences in infection loads between single‐ and double‐infected hosts for both parasites. These results suggest no strong interactions between the two parasites in honey bees, possibly due to spatial separation in the host. The significant positive correlation between *L. passim* and *N. ceranae* infection loads in double‐infected hosts therefore most likely results from differences among individual hosts rather than cooperation between parasites. Even if hosts are infected by multiple parasites, this does not necessarily imply that there are any significant interactions between them.

## 1 Introduction

Host–pathogen interactions are a major driving force of evolution and have received considerable attention (Olive & Sassetti, 2016; Rau et al., 2015; Woolhouse, Webster, Domingo, Charlesworth, & Levin, 2002). Although hosts infected by more than one parasite are common (Bordes & Morand, 2011), further attention is required to address interactions among such parasites within one host. These parasite–parasite interactions in individual hosts can potentially range from competition to cooperation (Alizon & Lion, 2011; Dobson, 1985; Griffin, West, & Buckling, 2004; Poulin, 2001; Read & Taylor, 2001; West & Buckling, 2003) and may have tremendous effects. For example, a host may cope with a single infection, but succumb to multiple ones, depending on pathogen–pathogen interactions (Neumann, Yañez, Fries, & de Miranda, 2012; Shen, Yang, Cox‐Foster, & Cui, 2005). This creates demand for better understanding the parasite–parasite interface in single hosts.

The health of western honey bees, *Apis mellifera*, has recently received considerable attention due to major losses of managed colonies at a global scale (Neumann & Carreck, 2010). Honey bee health is menaced by multiple stressors acting together or alone (Neumann & Carreck, 2010; Potts et al. 2010; Williams et al., 2010), with interactions among parasites likely to play a key role (Neumann et al., 2012).

**Figure 1:**
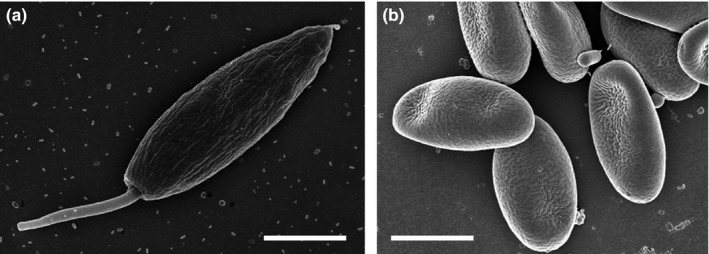
Scanning electron microscope images of the two honey bee parasites: (a) *Lotmaria passim*; lanceolate promastigote cell with anterior flagellum and caudate posterior extension visible (×10,000), (b) *Nosema ceranae*: ovocylindrical, straight to slightly curved spores (×10,000). Bars = 2 μm

The microsporidian gut parasite *N. ceranae* (Figure 1) switched hosts from Asian honey bees (*Apis cerana*) to *A. mellifera* (Fries, 2010), and appears to have replaced the endemic European *Nosema apis* in many areas (Fries et al., 2013). Analyses of archived samples revealed that *N. ceranae* has been present in western honey bees for several decades (Chen, Evans, Smith, & Pettis, 2008; Invernizzi et al., 2009; Paxton, Klee, Korpela, & Fries, 2007; Stevanovic et al., 2011), without replacing *N. apis* (Fernández et al., 2012; Forsgren & Fries, 2013; Martín‐Hernández et al., 2012). First reports of both microsporidian gut parasites concurrently infecting a single host suggest competition between *N. apis* and *N. ceranae* (Natsopoulou, Mcmahon, Doublet, Bryden, & Paxton, 2015; Williams, Shutler, Burgher‐MacLellan, & Rogers, 2014), but no competitive advantage was affirmed for *N. ceranae* in mixed infections for either infectivity or spore growth (Forsgren & Fries, 2010; Milbrath et al., 2015).

Recently, *Lotmaria passim*, a trypanosome intestine parasite of honey bees, has received attention (Figure 1; Ravoet et al., 2015; Schwarz et al., 2015; Arismendi, Bruna, Zapata, & Vargas, 2016). Whereas the two trypanosomatids, *Crithidia mellificae* and *L. passim*, can both infect honey bee colonies, *L. passim* is currently the predominant trypanosomatid in *A. mellifera* host populations globally (Schwarz et al., 2015). Based on accessioned sequences, all previous field data from honey bees in China, Italy, Japan, Spain, Switzerland, Turkey, and the USA were identified to be *L. passim*, and not *C. mellificae* as earlier suspected (Schwarz et al., 2015). Despite its global distribution, *L. passim* is poorly understood. However, it is known from laboratory experiments that mixed‐species infections with *C. mellificae* and *N. ceranae* can significantly affect local and systemic immune gene transcription within honeybees (Schwarz & Evans, 2013). Such altered immune responses may also occur during mixed‐species infections with *L. passim* and *N. ceranae*, but may differentially impact parasite populations.

As laboratory tests may not necessarily reflect field conditions (Retschnig et al., 2015), we took advantage of natural occurring infections of honey bee hosts with *N. ceranae* and *L. passim* in the field to test the following hypotheses: If the two parasites significantly interact with each other, we expect less or more individual hosts infected with both parasites compared to a random distribution. Likewise, infection loads of bees with one parasite alone should differ from those bees infected with both parasites. Lastly, positive or negative correlations between infection loads with both parasites are expected in double‐infected hosts given that they interact with each other.

## 2 Materials and Methods

### 2.1 Experimental setup

**Figure 2:**
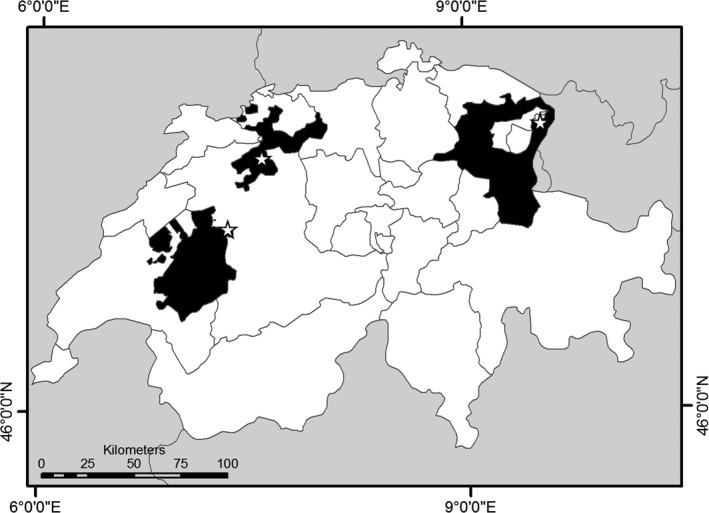
Map of Switzerland with Kantonal borders. The sampled Kantons are in black (from left to right: Fribourg, Solothurn and St. Gallen). Stars indicate apiary locations

In April 2011, a total of 408 adult honey bee workers were sampled from the outer frames of four queenright *A. mellifera* colonies at each of three apiaries in the Swiss cantons of St. Gallen, Fribourg and Solothurn (*N* = 12 colonies total, Figure 2). All samples were transported on ice to the laboratory (Dainat, Evans, Chen, & Neumann, 2011; Human et al., 2013) and stored at −20°C until further analyses.

### 2.2 DNA extraction

All sampled workers were crushed individually in 2‐ml microcentrifuge tubes containing 5 mm metal beads and 200 μl TN buffer (10 mmol/L Tris, 10 mmol/L NaCl; pH 7.6). The samples were homogenized with a TissueLyser for 30 s at 25 1/s frequency using a Qiagen Retsch^®^ MM 300 mixer mill (Evans et al., 2013). Then, the homogenates were centrifuged at 2500 rpm and 50 μl of the supernatant was used for DNA extraction using innuPrep DNA Mini Kit (Analytik Jena, Jena, Germany) following the manufacturer's recommendations. After DNA extraction, a Spectrophotometer Thermo Scientific™ NanoDrop 2000 full‐spectrum ultraviolet was used to quantify the DNA of each individual sample.

### 2.3 Classical and quantitative PCR

PCR assays were performed to detect the occurrence of microsporidians (*N. apis* and *N. ceranae*) and trypanosomes (*C. mellificae* and *L. passim*). The PCR analyses were performed by using MyTaq™ kit (Bioline) with 2 μl tenfold‐diluted DNA. We followed the manufacturer's recommendations by adding 5× reaction buffer, forward and reverse primers (final concentration of 0.4 μmol/L each) and 0.125 μl (0.63 Units) of Taq polymerase in a total of 25 μl final reaction volume. Three sets of species‐specific primers available for *Nosema* species (multiplex PCR primer combination Mnapis‐F, Mnceranae‐F, and Mnuniv‐R) and trypanosomes *C. mellificae* and *L. passim* (GAPDH‐F/R and Cr ITS1‐IR1/5.8R) were used (see Table 1). An independent PCR assay was performed for each set of primers. The qPCR cycling protocol was the same for each set of primers, which was as follows: cycling profile for all runs consisted of a 2‐min initial incubation at 95°C and 35 cycles of 20 s at 95°C for denaturation, 20 s at 56°C for annealing, 30 s at 72°C for extension, and finally 2 min at 72°C before the samples remained at 4°C to cool. The PCR products were qualitatively analyzed with a 2% two‐dimensional agarose gel electrophoresis. Each gel contained negative and positive controls and an appropriate DNA size standard. The gels were run for 45 min in the electrophoresis chamber containing 0.5 TBE (Tris–boric acid–EDTA) buffer. Subsequently to the electrophoresis, each gel was placed for 20 min in a GelRed™ bath for staining and visualization under ultraviolet light.

**Table 1:** Primers used for PCR diagnosis of microsporidian and trypanosome parasites in individual honey workers, *Apis mellifera*

Target	Primer	Sequence (5′–3′)	Size [bp]	Reference
*Nosema apis*	Mnapis‐F	GCA TGT CTT TGA CGT ACT ATG	224	Fries et al. (2013)
	Mnuniv‐R	GAC TTA GTA GCC GTC TCT C		
*Nosema ceranae*	Mnceranae‐F	CGT TAA AGT GTA GAT AAG ATG TT	143	Fries et al. (2013)
	Mnuniv‐R	GAC TTA GTA GCC GTC TCT C		
*Crithidia mellificae / Lotmaria passim*	GAPDH‐F	GTG CTC GTG GTG AAC GGC CA	402	B. Dainat, unpublished
	GAPDH‐R	GTC CTT GAG CGA CAC GCC GT		
	Cr‐ITS1‐IR1	GCT GTA GGT GAA CCT GCA GCA GCT GGA TCA TT	~1,200 to 1,500	Maia Da Silva et al. (2004); Schwarz et al. (2015)
	Cr‐ITS1‐5.8R	GGA AGC CAA GTC ATC CAT C		

The detected parasites were quantified by independent qPCR using KAPA SYBR^®^ FAST qPCR Master Mix (Kapa Biosystems) with 10 ng extracted DNA, 0.24 μl of forward and reverse specific primers (10 μmol/L) (Table 2) and 6 μl of 2× reaction buffer in a total of 12 μl final reaction volume (de Miranda et al., 2013). The qPCR cycling profile was the same for each set of primers and consisted of 3‐min incubation at 95°C and 40 cycles of 30 s at 95°C for denaturation, 30 s at 57°C for annealing, and a final extension at 50°C before cooling down to 4°C. Purified PCR products of known concentrations were used as standard curves on each individual plate, along with nontemplate controls and β‐Actin as reference gene (Scharlaken et al., 2008). Cq cutoff value (according to the value of the negative control) was used to define the disease status (positive or negative).

**Table 2:** Primers used for qPCR of *Nosema ceranae* and *Lotmaria passim* in individual honey bee workers, *Apis mellifera*

Target	Primer	Sequence (5′–3′)	Size [bp]	Reference
*Nosema ceranae*	Ncer bour F	AAG AGT GAG ACC TAT CAG CTA GTT G	104	Bourgeois et al. (2010)
	Ncer bour R	CCG TCT CTC AGG CTC CTT CTC		
*Lotmaria passim*	qCrFw1	TCC ACT CTG CAA ACG ATG AC	153	Runckel et al. (2011)
	qCrRev1	GGG CCG AAT GGA AAA GAT AC		
*A. m*. Actin	q92F	CGT TGT CCC GAG GCT CTT T	66	Gauthier et al. (2011)
	q157	TGT CTC ATG AAT ACC GCA AGC T		

### 2.4 Sequencing

Use of primers mentioned above requires subsequent sequencing of PCR products if the trypanosomatid species should be identified. Therefore, PCR products from selected samples were sequenced using Cr‐ITS1‐IR1/Cr‐ITS1‐5.8R primers (Table 1). Additionally, selected PCR products from the *N. ceranae* assays were also sequenced using Mnceranae‐F/Mnuniv‐R primers (Table 1). *N. ceranae* and *L. passim* were confirmed as the parasites present in our samples using reference sequences deposited in GenBank.

### 2.5 Data analyses

*Lotmaria passim* and *N. ceranae* parasites/bee were calculated following Bourgeois, Rinderer, Beaman, & Danka (2010) by comparing experimental Cq‐values with those of the standard curve. The calculation was performed by conversion factors from copies/μl to spore or cell equivalents/bee as follows:

$$\frac{\text{Number individual parasite}}{\text{bee}}=\frac{\left(\frac{\alpha \;\text{parasite copies}}{\mu l\;\text{PCR}}\right)\times \left(\frac{25\;\mu l}{0.05\;\text{bee}}\right)}{\beta \;\text{copies per genome}}$$

where α = copy number from real‐time PCR, β = 1 or 10 copies per genome for *L. passim* or *N. ceranae*, respectively, and 0.05 bee represents the 50 μl supernatant taken from the homogenate.

Data were tested for normality by using Kolmogorov–Smirnov tests. If data were normally distributed (Kolmogorov–Smirnov test, *p < *.05), groups were compared using a one‐way ANOVA. However, if normality was rejected (Kolmogorov–Smirnov test, *p > *.05), groups were compared using a Kruskal–Wallis ANOVA. In both cases, post hoc comparisons were made using Dunn's test. A Pearson correlation between *L. passim* and *N. ceranae* cell or spore equivalents was performed for those bees, which were infected by both parasites. We used a Chi‐square test to assess for significant differences between the observed and expected frequencies. Significant differences in the proportions of infected individuals among apiaries were analyzed using simple logistic regression. All statistical analyses were performed using the program NCSS (NCSS 9 Statistical Analysis and Graphics).

## 3 Results

From the 408 workers investigated, 21.57% (*N *=* *88) were naturally infected with *N. ceranae*, 12.99% (*N *=* *53) with *L. passim*, 1.96% (*N *=* *8) with both parasites and 63.48% (*N *=* *259) were not infected. Neither *C. mellificae* nor *N. apis* were found in any of the analyzed workers. The number of infected bees is significantly different from a random distribution, with *N. ceranae* being significantly more often found (Chi‐square test, χ^2^ = 4.324, df = 1, *p *<* *.05) than any other infection scenario. Furthermore, our data show no significant differences between observed and expected frequencies (i.e., bees infected with both parasites are not less common than expected from a random distribution) (Table 3). Differences in the proportion of gut parasite‐infected workers appeared among the apiaries. *N. ceranae* was detected significantly more often in bees from St. Gallen (97.68 ± 2.58–6.58) and Solothurn (143.77 ± 2.96–6.98) compared to Fribourg (*p *<* *.001, odds ratio ± 95% confidence interval). In contrast, significantly fewer bees were infected with *L. passim* in St. Gallen (0.14 ± −2.9–(‐)0.98) and Solothurn (0.32 ± −1.93–(‐)0.32) compared to Fribourg (*p *<* *.01, odds ratio ± 95% confidence interval). Honey bees not infected with parasites occurred significantly more in Fribourg than St. Gallen (0.43 ± −1.34–(‐)0.36) and Solothurn (0.26 ± −1.89–(‐)0.83) (*p *<* *.001, odds ratio ± 95% confidence interval). However, no significant differences were found for bees infected with both parasites among the three locations (*p *>* *.05) (see Table 4).

**Table 3:** Frequencies of individual honey bee workers (*Apis mellifera)* infected with *Lotmaria passim*,* Nosema ceranae* or both parasites. Observed frequencies (*O*), calculated expected frequencies (*E*), Chi‐square values, and respective *p*‐values are shown

Infections	*O*	*E*	(*O‐E*)^2^	(*O‐E*)^2^/*E* Chi‐square	*p*‐value
*L. passim* (yes) and *N. ceranae* (yes)	8	14.35	40.3225	2.81	>.05
*L. passim* (yes) and *N. ceranae* (no)	53	46.65	40.3225	0.86	>.2
*L. passim* (no) and *N. ceranae* (yes)	88	81.65	40.3225	0.49	>.2
*L. passim* (no) and *N. ceranae* (no)	259	265.35	40.3225	0.15	>.2

**Table 4:** Adjusted odds ratio for each parasite compared between the three different regions. Significant differences in proportions of infections levels between the regions are indicated with a (*)

Location	Odds ratio	Confidence interval	*p*‐Value
Honey bees infected with *Nosema ceranae*			
Fribourg: St. Gallen	97.68	2.58 to 6.58	.00001*
Fribourg: Solothurn	143.77	2.96 to 6.98	.00001*
Solothurn: St. Gallen	0.687	−0.93 to 0.16	.16493
Honey bees infected with *Lotmaria passim*			
Fribourg: St. Gallen	0.14	−2.9 to (−)0.98	.00007*
Fribourg: Solothurn	0.32	−1.93 to (−)0.32	.00599*
Solothurn: St. Gallen	0.44	−1.97 to 0.33	.16275
Honey bees infected with both parasites			
Fribourg: St. Gallen	7.05	−0.21 to 4.11	.07631
Fribourg: Solothurn	3.81	−1.08 to 3.75	.27748
Solothurn: St. Gallen	1.85	−1.05 to 2.28	.46784
Honey bees not infected with parasites			
Fribourg: St. Gallen	0.43	−1.34 to (−)0.36	.00063*
Fribourg: Solothurn	0.26	−1.89 to (−)0.83	.00001*
Solothurn: St. Gallen	1.65	−0.03 to 1.03	.0626

**Figure 3:**
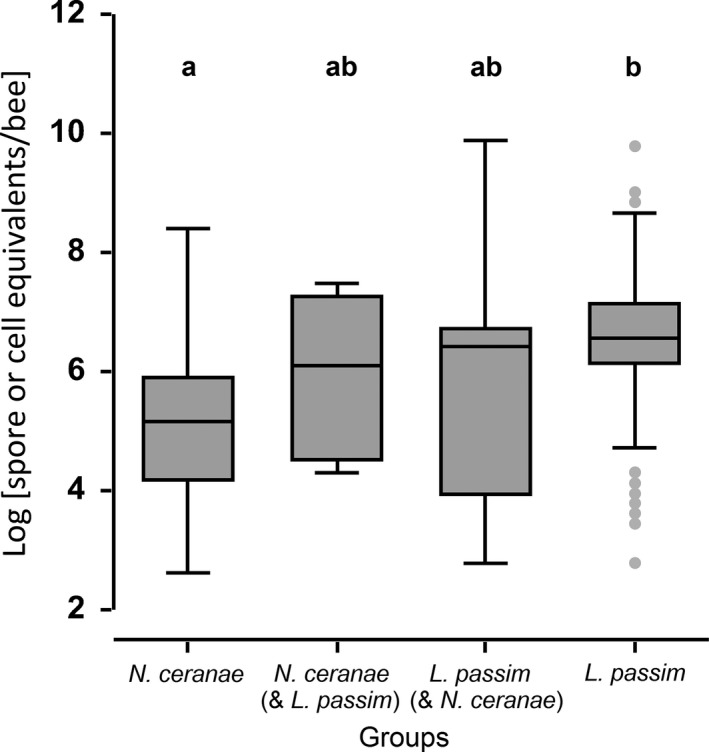
Infection levels of individual honeybee workers, *Apis mellifera*, with *Nosema ceranae* (*N* = 88), *Lotmaria passim* (*N* = 53) or both parasites (*N* = 8). Data are shown as spore or cell equivalents/bee at a log‐scale and display significant differences between *N. ceranae* and *L. passim*. No significant differences were found between bees infected with both parasites *N. ceranae* (& *L. passim*) and *L. passim* (& *N. ceranae*) and to single infected, respectively (means ± standard errors). All boxplots show the interquartile range (box), the median (black line within box), data range (horizontal black lines from box), and outliers (gray dots). Significant differences (Kruskal–Wallis ANOVA, Dunn's test, *p < *.0001) are indicated by letters (a,b)

**Figure 4:**
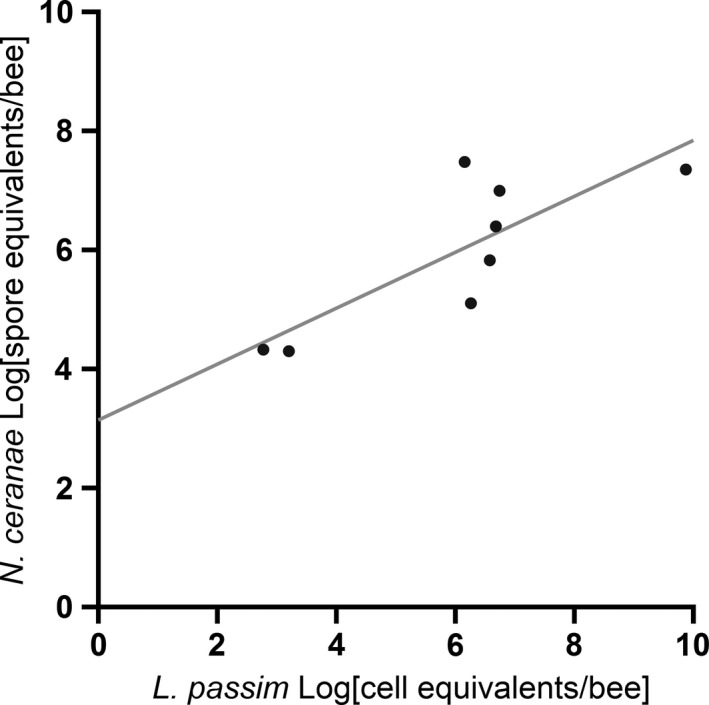
Infection levels of individual honeybee workers, *Apis mellifera*, with both *Nosema ceranae* and *Lotmaria passim*. A significant positive correlation was found (*n* = 8, Pearson |*r*| = .81, *p = *.015)

Overall, there were highly significant differences in infection levels (spore or cell equivalents/bee) between *N. ceranae* and *L. passim* (one‐way ANOVA, Dunn's test *p *<* *.0001, Figure 3). *L. passim* showed significantly higher infections levels compared to *N. ceranae*. However, there were no significant differences in infection levels for bees infected with both parasites and bees infected by one parasite alone or by both parasites comparing the same species (one‐way ANOVA, Dunn's test *p *>* *.05, Figure 3). In bees which were infected with both parasites, a significant positive correlation was found between infection loads of the two parasites (Pearson correlation: Pearson |*r*| = .81, *p *=* *.015; Figure 4).

## 4 Discussion

The results of this study suggest little to no interaction between the two honey bee parasites *L. passim* and *N. ceranae*. This is possibly due to spatial separation in the naturally infected hosts (Higes, García‐Palencia, Martín‐Hernández, & Meana, 2007; Schwarz et al., 2015). The observed positive correlation between *L. passim* and *N. ceranae* infection loads in double‐infected workers most likely reflects differences among individual hosts, for example due to genetics, food and/or age.

Neither *C. mellificae* nor *N. apis* were found in any of the analyzed workers. This confirms earlier studies that *N. apis* is currently rare in Switzerland (Dainat, Evans, Chen, Gauthier, & Neumann, 2012; Retschnig, Williams, & Neumann, in revision), and also supports that *L. passim* is currently the predominant trypanosomatid in *A. mellifera* host populations (Schwarz et al., 2015). Conversely, infections with both *L. passim* and *N. ceranae* were equally common in all three apiaries, thereby confirming the widespread occurrence of these two honey bee parasites (Fries, 2010; Ravoet et al., 2013, 2015; Schwarz et al., 2015). A higher number of workers infected with *N. ceranae* were found in Solothurn and St. Gallen compared to those from Fribourg. Similarly, infections with *L. passim* were more often found in Fribourg compared to the other two locations. However, no significant differences were seen among the apiaries when individual bees were infected with both parasites. The underlying reasons for these observed differences in infections remain unclear.

As both *L. passim and N. ceranae* are common parasites of the honeybee (Schwarz et al., 2015), the absence of both parasites in the majority of samples seems interesting. However, one has to take into account that we did analyze individual workers in this study. Indeed, recent surveys using pooled worker bee samples showed that 46.7% of Swiss honey bee colonies are infected with *Nosema* spp. (Retschnig et al., in revision) and 82.5% with *L. passim* (Schneeberger, Yañez, Retschnig, Williams, & Neumann, 2017), respectively. Therefore, the overall prevalence of the two parasites in Switzerland is well in line with earlier studies for other regions (e.g., Schwarz et al., 2015; Stevanovic et al., 2011, 2016).

The data show no significant differences between the expected and observed frequencies of single‐ and double‐infected bees. We therefore have no evidence that these two parasites interfere with each other's chances of infecting a host, pointing into the direction of no or weak parasite–parasite interactions. Similarly, a fairly high rate of honey bee colonies (60.5%) was coinfected with *L. passim* and *N. ceranae* over a 9 years survey, but no detectable correlation was found between the rates at which the two parasite‐infected colonies (Stevanovic et al., 2016).

The observed infection loads of individual bees were significantly higher for *L. passim* compared to *N. ceranae*, and are well within the limits of previous reports for foraging bees (Bourgeois et al., 2010; Ravoet et al., 2013, 2015; Retschnig, Neumann, & Williams, 2014; Retschnig et al., 2015). Despite the observed high infection loads, there were no significant differences between the infection loads of both parasites in single‐ or double‐infected hosts. Again, this provides no support for either competition or cooperation between the two parasites, even though single‐ and mixed‐species *C. mellificae* trypanosome and *N. ceranae* microsporidia infections elicit distinct immune responses (Schwarz & Evans, 2013).

So, why are there no significant interactions between the two intestine parasites *N. ceranae* and *L. passim* despite high infection loads? While *N. ceranae* has been detected using PCR in other tissues than the gut (i.e., hypopharyngeal glands, salivary glands, Malpighian tubules, and fat body, Chen et al., 2009), this microsporidian only develops spores in the midgut (Higes et al., 2007). However, *L. passim* prefers to colonize the rectum tissue of honey bees (Schwarz et al., 2015). This spatial separation in infected hosts may explain why the interactions between the two parasites are not significant, because they actually might not compete for the same niche. However, on the other hand, the significant positive correlation between *L. passim* and *N. ceranae* infection loads in concurrently infected hosts may indicate some cooperation between the parasites (Alizon & Lion, 2011).

Genetically based differences in honey bee susceptibility to pathogens are long known (Rothenbuhler, 1964) and have also been found for *N. ceranae* (Huang et al., 2014). Moreover, honey bee colonies consist of many different subfamilies (patrilines) due to high degrees of polyandry of the queens (Neumann, Moritz, & van Praagh, 1999). It is therefore extremely likely that honey bee workers even from the very same colony can differ in their susceptibility to parasites due to genetics alone, thereby offering an alternative explanation for the observed positive correlation between infection loads given that some hosts are more vulnerable to both parasites compared to others (i.e., higher or lower infection loads for both parasites, respectively). Alternatively, environmentally imposed differences among individual hosts can also explain this trend. For example, protein fed to honey bee workers can increase *N. ceranae* spore numbers (Jack et al., 2016), and some workers may have consumed more pollen than others. Finally, it is known that age can play a key role for *N. ceranae* spore loads of honey bees (Smart & Sheppard, 2012). Therefore, the observed significant correlation between infections loads of the two parasites *L. passim* and *N. ceranae* can be parsimoniously explained without assuming any interactions whatsoever between the two parasites. In light of our other results and our random sampling of bees, we consider it more likely that differences among hosts are underlying the observed positive correlation between *L. passim* and *N. ceranae* infection loads. In any case, correlation is obviously not causation, and further experiments with bees of both known age and pollen consumption under single and double infection scenarios are required to understand the underlying mechanisms for the observed correlation.

In conclusion, our data do not suggest any strong interactions between the two honey bee parasites, which may be explained by spatial separation in the host.

## Conflict of Interest

None declared.
